# The use of ^68^Ga prostate-specific membrane antigen PET-CT in prostate cancer: diagnostic challenges and therapeutic opportunities

**DOI:** 10.2144/fsoa-2021-0035

**Published:** 2021-04-07

**Authors:** Elisena Franzese, Stefano De Falco, Maria Maddalena Laterza, Liliana Montella, Sergio Facchini, Carmela Liguori, Paola Coppola, Ylenia Diessa, Massimiliano Berretta, Salvatore Pisconti, Francesco Trama, Matteo Ferro, Felice Crocetto, Morena Fasano, Carlo Buonerba, Gaetano Facchini

**Affiliations:** 1 Medical Oncology Complex Unit, ASL Napoli 2 Nord ‘Santa Maria delle Grazie’ Hospital, Pozzuoli, Italy; 2 Department of Precision Medicine, University of Campania ‘Luigi Vanvitelli’, Napoli, Campania, Italy; 3 Unit of Infectious Diseases, Department of Clinical & Experimental Medicine, University of Messina, Messina, Italy; 4 Oncology Unit, SG Moscati Hospital, Taranto, Italy; 5 Andrology & Urogynecological clinic, Santa Maria Terni Hospital, Terni, University of Perugia, Perugia, Italy; 6 Division of Urology, European Institute of Oncology, Milan, 20141, Italy; 7 Department of Neurosciences, Human Reproduction & Odontostomatology, University of Naples Federico II, Naples, Italy; 8 Department of Oncology & Hematology, Regional Reference Center for Rare Tumors, Department of Oncology & Hematology, AOU Federico II of Naples, Naples, 80131, Italy; 9 National Reference Center for Environmental Health, Zoo-prophylactic Institute of Southern Italy, Portici, 80055, Italy

**Keywords:** nuclear medicine, prostate cancer, PSMA PET

Prostate cancer is one the most commonly diagnosed malignancies in men, with a total of 1,414,259 new cases and 375,000 deaths estimated worldwide in 2020 [1]. Although several novel pharmacologic agents, including chemotherapy, hormonal, radiopharmaceutical and biological drugs [2], have become part of the therapeutic armamentarium against metastatic prostate cancer, advanced disease still represents a deadly condition in virtually all patients. The discovery of novel prostate-specific antigens has yielded significant progresses in both the therapeutic strategy and imaging techniques. Prostate-specific membrane antigen (PSMA) is a transmembrane protein that is expressed in normal and neoplastic prostate tissue, with a structure composed of a 707-amino-acid external portion, a 19-amino-acid internal portion, a 24-amino-acid transmembrane portion [3]. In light of its specificity, PSMA has been selected as the biological target of a number of radiolabeled small molecules, such as [^68^Ga]-PSMA-11, [^18^F]-DCFPyL and [^18^F]-PSMA-1007 [4]. While the standard of care for imaging of prostate cancer to assess stage and response to treatment continues to be based on conventional imaging, including whole-body bone scans, abdominopelvic computed tomography (CT) and MRI [5], prostate-specific membrane antigen positron emission tomography (PSMA-PET) has definitively proven to be a highly accurate staging tool in multiple settings, although its exact uses in clinical practice remain to be determined.

A recently published trial designed to assess PSMA-PET accuracy compared with standard imaging to detect pelvic nodal or distant-metastatic disease was conducted in 302 men with high-risk, localized prostate cancer, randomized to imaging with CT+ bone scan followed by [^68^Ga] PSMA-11 PET-CT or vice versa [6]. In the subgroup of 295 (98%) men with adequate follow-up, 87 (30%) showed distant metastatic or pelvic nodal disease. Compared with conventional imaging, not only did PSMA PET-CT show a 27% (95% CI: 23–31) higher overall accuracy (92% [88–95] vs 65% [60–69]; p < 0.0001), with a higher sensitivity (85% [74–96] vs 38% [24–52]) and specificity (98% [95–100] vs 91% [85–97]), but it also led to more frequent changes in the treatment plan (41 [28%] men [21–36] vs 23 [15%] men [10–22]; p = 0.008), with less frequent equivocal findings (7% [4–13] vs 23% [17–31]). PSMA PET has also proven to be highly accurate in detecting disease in men with biochemical recurrence. In a single-arm prospective study including 635 men assessed using [^68^Ga]-PSMA-11 PET because of biochemical recurrent disease detected after undergoing radiation therapy (n = 169 [27%]), prostatectomy (n = 262 [41%]) or both (n = 204 [32%), a positive predictive value using histology as gold standard of 0.84 (95% CI: 0.75–0.90) was reported, with detection rates varying according to prostate specific antigen (PSA) levels (38% for <0.5 ng/ml [n = 136]; 57% for 0.5 to <1.0 ng/ml [n = 79]; 84% for 1.0 to <2.0 ng/ml [n = 89; 86% for 2.0 to<5.0 ng/ml [n = 158] and 97% for ≥5.0 ng/ml [n = 173; p < 0.001]). These results have been confirmed in a large meta-analysis which included 29 trials in the quantitative analysis and showed that [^68^Ga]-PSMA-11 yielded a specificity and sensitivity of 0.96 (95% CI: 0.85–0.99) and 0.74 (95% CI: 0.51–0.89), respectively, for assessment of nodal disease compared with histology used as gold standard. Furthermore, in men with biochemical recurrence the positive predictive value of PSMA-PET was 0.99 (95% CI: 0.96–1.00) [8], although it is likely to be lower in men with solitary lesions, especially in the ribs [9]. Additional relevant clinical information regarding the expected course of the disease may be provided by measuring wholebody and tumor metabolic tumor volume on PSMA-PET, with a higher volume indicative of a more aggressive disease [10,11], while no PSMA tracer on the market seems to be more reliable than the other, although superiority of PSMA versus choline has been established [12].

The high accuracy and versatility of PSMA-PET in various clinical settings have brought multiple key questions in clinical practice. First of all, it is unknown whether PSMA-PET may provide additional useful information to detect high-volume disease compared with the definition based on conventional imaging used in the CHAARTED criteria are based on conventional imaging as opposed to PSMA PET. Can PSMA PET provide addition info to define high-volume disease? [13]. Given the potential predictive value of tumor volume not only in patients receiving docetaxel but also in those receiving androgen receptor axis-targeted (ARAT) agents [14], PSMA-PET may represent an excellent tool to improve detection of high-volume disease in the setting of metastatic castration sensitive prostate cancer. Conversely, diagnosis of oligo-metastatic disease [15] that may benefit from stereotactic radiotherapy of metastatic lesions [16] may be truly more accurate using a highly sensitive technique such as PSMA-PET imaging. The effects of routine use of PET- PSMA imaging in clinical practice may be more profound in men who have been diagnosed with nonmetastatic castration-resistant prostate cancer and are, therefore, eligible for treatment with ARAT agents such as apalutamide, darolutamide and enzalutamide [17]. It may be argued that patients who are nonmetastatic using conventional imaging but appear to have metastatic disease on PSMA-PET may not be eligible to be treated with the ARAT agents approved in this setting, despite that these men would have been enrolled in the registrative trials. Furthermore, reimbursement may also be an issue. As some drugs, such as apalutamide and darolutamide, are only approved in men with nonmetastatic disease who are at risk of developing metastatic disease, but not in those with a demonstrated metastatic disease, the use of PSMA-PET compared with standard imaging may prevent access to these agents in some patients. Finally, additional questions regard the use of PSMA-PET in men with metastatic disease treated in advanced settings (e.g., with cabazitaxel [18,19]). Detection of progressive disease on PSMA-PET in such patients may lead to therapy change even in those who have clear clinical benefit (e.g., improvement in pain or performance status).

The advent of [^177^Lu]-PSMA-617, a radiopharmaceutical agent capable of delivering β radiation to PSMA-expressing cells has made PSMA-PET the ideal screening tool to detect men who are most likely to benefit from such an innovative treatment, as shown by the recently published TheraP trial [20].

PSMA-PET has great potential to improve staging of prostate cancer and expand treatment options for men with such a vicious disease. In summary, the most promising therapeutic implications include detection of metastatic disease in newly diagnosed male candidates for surgery, detection of oligometastatic disease in patient candidates for stereotactic body radiotherapy and assessment of eligibility for [^177^Lu]-PSMA-617 in men with advanced disease treated with multiple systemic agents.
